# Factors affecting pleural fluid adenosine deaminase level and the implication on the diagnosis of tuberculous pleural effusion: a retrospective cohort study

**DOI:** 10.1186/1471-2334-13-546

**Published:** 2013-11-16

**Authors:** Tunn Ren Tay, Augustine Tee

**Affiliations:** 1 Department of Respiratory Medicine, Changi General Hospital, 2 Simei Street 3, Singapore 529889, Singapore

**Keywords:** Adenosine deaminase, Tuberculosis, Pleural effusion, Biological markers

## Abstract

**Background:**

Adenosine deaminase (ADA) is useful in the diagnosis of tuberculous pleural effusion (TPE). This study aims to determine the factors affecting pleural fluid ADA levels and to establish the optimal ADA levels for diagnosis of TPE for different age groups.

**Methods:**

This was a retrospective study from January 2007 to October 2011. One hundred and sixty patients who had pleural fluid ADA performed for investigation of pleural effusion were analyzed. Variables examined included demographics, pleural fluid characteristics and peripheral blood counts. The ADA cut-offs according to age were selected using the receiver operating characteristic (ROC) curve.

**Results:**

The mean pleural fluid ADA was significantly higher in the TPE group (100 ± 35 IU/L) compared to non TPE patients (30 ± 37 IU/L). There was significant correlation between pleural fluid ADA and age, pleural fluid protein, LDH, and fluid absolute lymphocyte count. The strongest correlation was seen with age (r = −0.621). For patients ≤ 55 years old the ROC for ADA had area under curve (AUC) of 0.887. A pleural fluid ADA of 72 IU/L had sensitivity of 95.1%, specificity of 87.5%, positive predictive value (PPV) of 95.1% and negative predictive value (NPV) of 87.5% for the diagnosis of TPE. For patients > 55 years old the AUC is 0.959. ADA of 26 IU/L had a sensitivity of 94.7%, specificity of 80.4%, PPV of 62% and NPV of 97.8%.

**Conclusions:**

There is a significant negative correlation between pleural fluid ADA and age. For older patients, a lower ADA cut-off should be used to exclude TPE.

## Background

Pleural effusion is a common medical condition with many possible underlying etiologies. Neutrophilic predominant exudative effusions are due to acute processes e.g. pneumonia or acute pulmonary embolism [1] whereas lymphocytic effusions have a much longer list of differential diagnoses [2]. However, in areas with high incidence of tuberculosis (TB), pleural tuberculosis and malignancy are the most likely cause of a lymphocytic pleural effusion [3,4].

Pleural fluid analysis and closed pleural biopsy are integral parts in the investigative work up of an exudative pleural effusion. For diagnosis of tuberculous pleural effusion (TPE), the yield of pleural fluid culture for mycobacteria is low at about 36% [5]. Joint sensitivity of biopsy tissue samples is as high as 90% [5], but closed pleural biopsy is a relatively invasive procedure and involves a long waiting time for mycobacteria culture results. Pleural fluid adenosine deaminase (ADA) has thus become an important diagnostic tool in the evaluation of exudative pleural effusions because it is inexpensive, rapid and has a high accuracy with sensitivity and specificity of up to 100% and % respectively for diagnosis of TPE [6].

Adenosine deaminase (ADA) is an enzyme which catalyses the conversion of adenosine to inosine and plays an important role in the differentiation of lymphoid cells. Its activity is high in diseases in which cellular immunity is stimulated [1]. Different cut off values of ADA ranging from 30–100 IU/L have been used in various studies, with differing sensitivities and specificities [7]. The discrepancies in the results can be due to different methods of ADA analysis, prevalence of TB in various study populations as well as differences in study population characteristics. Previous studies have demonstrated correlation between pleural ADA level and CD4 lymphocyte counts [8], as well as lower mean ADA level in Japanese patients with TPE [9]. This suggests that patient immune status as well as demographic factors may affect pleural fluid ADA levels.However, few studies have looked into factors which can affect pleural fluid ADA levels.

Our study therefore aims to:

a) Determine whether certain patient demographic characteristics, pleural fluid biochemistry and peripheral blood leucocyte counts affect pleural fluid ADA level.

b) Establish ADA cut off value for diagnosis of TPE in our study population.

If pleural fluid ADA does correlate significantly with the variables studied, ADA value for patients should then be interpreted based on patient demographics and relevant laboratory investigations.

## Methods

This was a retrospective study of the medical records of patients investigated for pleural effusion in our hospital from January 2007 to October 2011. The study design was approved by the Institutional Review Board (IRB) and exempted from further IRB review. Based on laboratory records a list of consecutive patients with pleural fluid ADA sent was obtained. We felt that this was a reliable way of obtaining the list of patients investigated for pleural effusion as pleural fluid ADA is routinely sent in our institution for all cases of pleural effusion. Pleural fluid ADA was measured by the spectrophotometric method described by Giusti and Galanti. 80 patients with TPE were identified. Another 80 patients with diagnoses other than TPE were randomly selected using a random number generator.

Pleural effusions were diagnosed to be due to pleural TB (TPE) if: (i) pleural fluid smear was positive for acid fast bacilli or fluid culture was positive for mycobacteria tuberculosis; (ii) pleural biopsy histology showed granulomas with no other causes of granulomatous lung disease; (iii) pleural biopsy culture positive for mycobacteria tuberculosis; (iv) sputum culture positive for mycobacteria tuberculosis. Malignant pleural effusions were confirmed by positive pleural fluid cytology or pleural biopsy histology (closed biopsy or medical thoracoscopically obtained). Parapneumonic effusions were diagnosed based on clinical and radiographic features that were consistent with an acute pulmonary infection and exclusion of other causes of pleural effusion. Effusions were attributed to congestive cardiac failure (CCF) if the effusion was a transudate with consistent clinical features. An effusion was considered to be lymphocyte-predominant if pleural fluid lymphocyte count was > 0.5 of total fluid leucocyte count, and neutrophil-predominant if pleural fluid neutrophil count was > 0.5 of total leucocyte count. Peripheral blood counts used for analysis were most recent to the date of pleural fluid investigations.

Continuous variables are expressed as mean ( SD) while categorical variables are expressed as number and group percentages. Differences in ADA levels between groups were analysed using unpaired Student t test and one way Anova. Correlation between ADA and specified variables was quantified using Pearson correlation coefficient. This was performed for the whole study population (n = 160) as well as individually for the TPE and non TPE groups. A correlation coefficient (r) of < 0.3 was considered to be negligible correlation, r 0.3-0.5 was considered weak correlation, r 0.5-0.7 was considered moderate correlation, r 0.7-0.9 was high correlation and r > 0.9 was very high correlation. The best cut off value of ADA for diagnosis of TPE was selected by receiver operating characteristics (ROC) curve. We also fitted a multivariate linear regression model and included all variables identified to be statistically significant to see which ones remain independently associated with ADA level. All statistical analyses were performed using IBM SPSS Statistics version 19. A 2 tailed p value of < 0.05 was taken to be statistically significant.

## Results

A total of 160 patients were investigated in this study. The baseline characteristics of these patients are shown in Table 1. 80 (50%) patients were diagnosed with TPE, 23 (14.4%) had malignant effusions, 40 (25%) had parapneumonic effusions, and 17 (10.6%) had effusions which were classified as ‘Others’. Of the 17 cases, 14 were attributed to congestive cardiac failure and 3 were undetermined. The 3 patients with undetermined effusions were followed up for up to one year on hospital discharge. 2 had complete resolution of the effusion with no recurrence whereas the third had residual pleural effusion but declined further investigations. Most of the patients were male (73.1%) and Chinese (64.4%). The mean age of the study population was 55 ± 21 yrs. The subgroup of TPE patients had a lower mean age with the majority (76.3%) age 55 yrs and below. This contrasted with the non TPE group who had a higher mean age and with only 30% age 55 yrs and below.

**Table 1:** Baseline characteristics

	**All (n = 160)**	**TPE (n = 80)**	**Non TPE (n = 80)**
**Gender**			
Male	117 (73.1%)	58 (72.5%)	59 (73.8%)
**Race**			
Chinese	103 (64.4%)	38 (47.5%)	65 (81.3%)
Malay	26 (16.3%)	16 (20%)	10 (12.5%)
Indian	15 (9.4%)	12 (15%)	3 (3.8%)
Others	16 (10%)	14 (17.5%)	2 (2.5%)
**Mean age (years)**	55 ± 21	44 ± 19	66 ± 17
**Number of patients with age ≤ 55 years**	85 (53.1%)	61 (76.3%)	24 (30%)
**Number of patients with age > 55 years**	75 (46.9%)	19 (23.8%)	56 (70%)
**Mean ADA level (IU/L)**	65 ± 51	100 ± 35	30 ± 37

Mean pleural fluid ADA was significantly higher in the TPE group compared to non TPE group (100 ±35 IU/L vs 30 ± 37 IU/L, p < 0.001). There was no statistically significant difference in pleural fluid ADA level between the malignant, parapneumonic and others group, with mean ADA 28 ± 49 IU/L, 40 ± 34 IU/L and 9 ± 6 IU/L respectively. Only 1 patient was HIV positive (0.6%), 88 were HIV negative (55%) and HIV status was unknown in 71 (44.4%).

There was no significant difference in pleural fluid ADA level between genders. The Chinese appeared to have a lower mean pleural ADA compared to the Indians and other races, with ADA of 52 ± 48 IU/L, 94 ± 47 IU/L, and 110 ± 48 IU/L respectively. However, the difference in ADA levels seen between the racial groups was likely due to the lower proportion of TPE in Chinese compared to the Indians and other races (36.9%, 80% and 87.5% respectively). This explanation was supported by subgroup analyses of TPE and non TPE patients which did not reveal any difference in ADA levels between the racial groups. There was a moderate negative correlation between age and pleural ADA, r = −0.621, p < 0.001, indicating that pleural fluid ADA decreases with age. There was also a moderate positive correlation between ADA and pleural protein, r = 0.556, p < 0.001. Weakly positive correlations were seen between pleural fluid ADA and pleural lactate dehydrogenase (LDH), r = 0.427, p < 0.001, and pleural absolute lymphocyte count, r = 0.314, p < 0.001. Peripheral blood white cell counts and lymphocytes count had negligible correlation with ADA with r = −0.177, p = 0.025 and r = 0.201, p = 0.011 respectively.

We proceeded to analyse pleural fluid ADA levels in the TPE and non TPE groups as shown in Table 2. In the TPE group, those age > 55 yrs had significantly lower pleural ADA levels than those ≤ 55 yrs. When analysed as continuous variablesthere was weak negative correlation between age and pleural fluid ADA with r = −0.281, p < 0.05. There was statistically significant correlation between ADA, pleural protein and pleural LDH, but not with pleural cell count and lymphocyte count.For non TPE patients, there was significant negative correlation between ADA and age, and positive correlation with pleural protein, LDH, cell count and absolute lymphocyte count.

**Table 2:** ADA analysis and correlation with study variables for different subgroups

	**All patients (n = 160)**		**TPE (n = 80)**		**Non TPE (n = 80)**	
**Gender**						
**Mean ADA**						
**(IU/L)**						
Male	66 ± 50	p = 0.712	98 ± 37	p = 0.364	35 ± 42	P = 0.067
Female	63 ± 52		106 ± 32		17 ± 16	
**Race**						
**Mean ADA**						
**(IU/L)**						
Chinese	52 ± 48	p < 0.001	95 ± 37	p = 0.306	27 ± 33	P = 0.121
Malay	73 ± 43		95 ± 36		38 ± 27	
Indian	94 ± 47		113 ± 28		19 ± 18	
Others	110 ± 48		110 ± 36		115 ± 95	
**ADA level**						
**According to age**						
**(IU/L)**						
Age ≤ 55 years	92 ± 47	P < 0.001	107 ± 33	P = 0.002	56 ± 57	P = 0.000
Age > 55 years	34 ± 34		79 ± 34		19 ± 15	
**Age corr coeff (r)**	r = −0.621	p < 0.001	r = −0.281	p = 0.011	r = −0.556	P = 0.000
**Fluid**						
**Corr coeff (r)**						
Protein	r = 0.556	p < 0.001	r = 0.442	p < 0.001	r = 0.343	P = 0.02
LDH	r = 0.427	p < 0.001	r = 0.473	p < 0.001	r = 0.836	P = 0.000
Cell count	r = 0.237	p = 0.003	r = 0.160	p = 0.162	r = 0.573	P = 0.000
Lymphocytes	r = 0.314	p < 0.001	r = 0.116	p = 0.305	r = 0.534	P = 0.000

Multivariate linear regression analysis was performed and we found that the independent predictors of pleural fluid ADA were age, pleural fluid protein, LDH, and absolute lymphocyte count.

**Figure 1:**
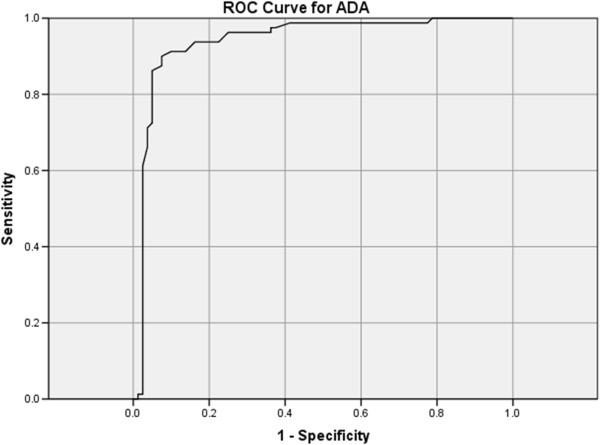
ROC curve for ADA.

The receiver operating curve (ROC) for ADA was performed for our study population. The area under curve (AUC) was 0.941 (95% CI 0.899-0.983) (Figure 1). ADA level of 45 IU/L would have a sensitivity of 93.8% and specificity of 82.5% for the diagnosis of TPE.We attempted to determine the optimal pleural fluid ADA level for the diagnosis of pleural TB for different age groups, taking age cut-off of 55 years old. Age cut-off of 55 years was taken because in clinical practice the suspicion of malignancy and malignant pleural effusion increases with age, especially after age 50 years. Cut off of 55 years would divide our study population into roughly equal numbers in the 2 age groups for comparison. Receiver operating characteristics (ROC) curves were described for both age groups.

**Figure 2:**
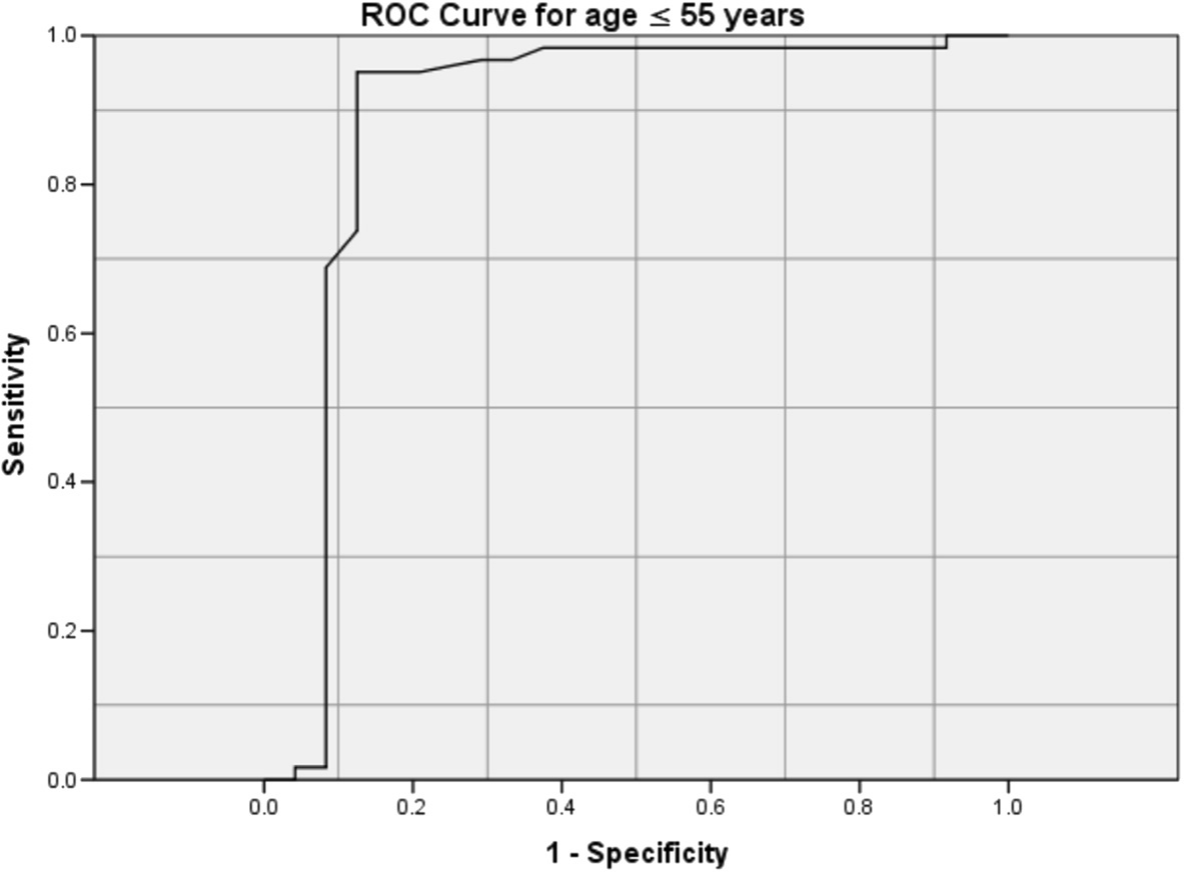
ROC curve for age ≤ 55 years.

**Figure 3:**
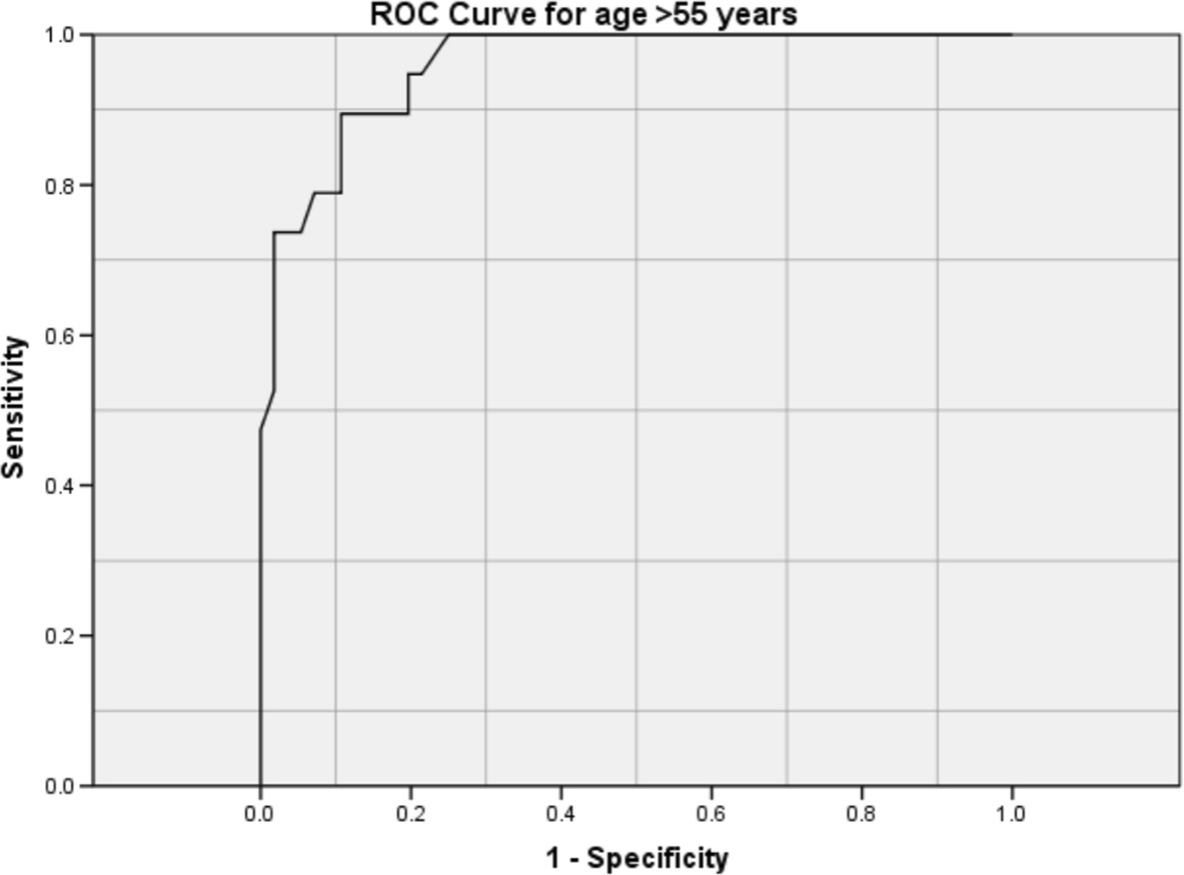
ROC curve for age > 55 years.

For patients age ≤ 55 yrs the ROC for ADA had AUC of 0.887 (95% CI 0.775-0.999) (Figure 2). A pleural fluid ADA of 72 IU/L would have a sensitivity of 95.1%, specificity of 87.5%, positive predictive value (PPV) of 95.1% and negative predictive value (NPV) of 87.5% for the diagnosis of TPE. For patients age > 55 yrs the ROC for ADA had AUC of 0.959 (% CI 0.918-0.999) (Figure 3). ADA of 26 IU/L would give a sensitivity of 94.7%, specificity of 80.%, PPV of % and NPV of 97.8% for the diagnosis of TPE. To improve specificity in this group of patients, a higher ADA cut-off of 46 IU/L would give a specificity of 92.9% but a resultant lower sensitivity of 78.%.

## Discussion

Our study suggests that patient and pleural fluid characteristics need to be considered during interpretation of pleural fluid ADA as it decreases with age and increases with pleural fluid protein. This is also, to the best of our knowledge, one of the first few studies to determine the ADA level for diagnosis of TPE according to different age groups: 72 IU/L in those age ≤ 55 yrs and 26 IU/L in those > 55 yrs.

This study adds to a relatively small number of papers published on pleural fluid ADA performed on an Asian population. Contrary to the study by Niwa et al. [9] which showed an ADA level of only 42.9 IU/L in patients with TPE and thus leading to a suspicion that ADA may be less useful in Asians, patients with TPE in our study had mean ADA of 100 IU/L which was similar to that reported by Riantawan et al. [10].

Few studies have looked at the factors affecting pleural ADA. In our study age had the strongest correlation with pleural fluid ADA. We noted that the correlation between age and ADA was much weaker when examined within the subgroup of TPE patients. One possible explanation is that the number of older patients aged > 55 years was relatively small in the TPE group resulting in a less representative population. The relationship between age and ADA demonstrated in our study was similar to that of Yeon et al. from Korea [11]. Their study showed a significantly higher ADA in patients with exudative pleural effusions who were older than 65 yrs compared to those less than 65 yrs and the ADA cut-off for the diagnosis of TPE in the older group was also much lower than for the younger group (25.9 IU/L compared to 49.1 IU/L). Other authors have also shown that young patients with TPE have a much higher level of pleural fluid ADA. In a group of patients age ≤ 35 yrs the mean ADA level for those with TPE was 111.1 IU/L, a similar figure to our study’s younger TPE group [12]. Merino studied a paediatric population (age < 18 yrs) with TPE and the mean ADA level obtained was 73.8 IU/L with all but 2 patients (%) having ADA less than 40 IU/L [13]. It may be possible that the decrease in ADA with age does not occur as a continuum throughout all ages but is evident only after a certain age.

Lee et al. [14] examined patients with non-tuberculous lymphocytic effusions and found a fairly positive correlation between ADA, pleural protein and LDH, similar to our findings. In the study by Kashiwabara et al. [15] which consisted of a larger proportion (%) of parapneumonic effusion and mainly non-lymphocytic exudates, there was only positive correlation between ADA and LDH, but no significant correlation with protein or age. Our study showed a poor correlation between ADA and pleural cell count, and no correlation with blood lymphocyte count. This was similar to findings in other studies [10,14,16]. In fact,other authors have shown that the sensitivity of ADA was not affected by the CD4 count in pleural fluid and was still useful diagnostically in HIV positive patients [10,16].

ADA has greatest activity in lymphoid tissues and is responsible for the differentiation of lymphoid cells. There are 2 isoenzymes, ADA1 and ADA2, with ADA2 found only in monocytes and macrophages. The high total level of ADA in tuberculous pleural effusion is due largely to high ADA2 activity [17]. There is biologic plausibility of the negative correlation between ADA and age, attributable to the phenomenon of immunosenescence [18]. There is increasing evidence that there is loss of immune function in the elderly individual. We noted a weaker correlation between ADA and age in the TPE subgroup compared to the overall study population. Apart from the possible effect due to a small sample size of elderly TPE patients mentioned earlier in the discussion, another postulation is that ageing may affect monocytes and macrophages to varying degrees compared to lymphocytes and subsequently a smaller effect on ADA2 isozyme production, which is the predominant isoenzyme in TPE. Pleural protein and LDH are both indicators of the degree of pleural inflammation [1] and there would be conceivably more activated lymphocytes and ADA production in the presence of greater pleural inflammation. Lee at al [11] previously offered an explanation for the lack of association between ADA and pleural cell count. The standard ADA determination measures ADA activity and not the absolute amount of enzyme present. ADA activity may be dependent more on the pathologic stimulus e.g. TB and rapidity of T lymphocyte proliferation, and not on amount of lymphocytes present.

One clinical application of our study’s findings would be the interpretation of pleural fluid ADA according to patient characteristics. Pleural fluid ADA decreases with age and therefore increases the number of ‘false negative’ results for diagnosis of TPE when a fixed cut-off level is used in an older population compared to a younger population. In our study, if the widely accepted standard ADA cut-off level of 50 IU/L was used, 5 of the 19 patients (26.%) with TPE in age group > 55 yrs would have a false negative result. If the cut-off level of 26 IU/L was used, only 1 patient (5.%) would have a false negative ADA result. Similarly, caution might have to be exercised in excluding a diagnosis of TPE based on low ADA level if the pleural protein and LDH are also low.

Limitations of the study include its retrospective nature but selection bias in this study would have been limited as all patients in our institution investigated for pleural effusion would have pleural fluid ADA performed. The etiologies of 3 of the pleural effusions were undetermined but none had an eventual diagnosis of TPE.

## Conclusions

In summary, we have demonstrated that pleural fluid ADA is affected by age and pleural fluid protein. Using a lower ADA level to exclude TPE in an older population can reduce the number of false negative results, and based on the pleural fluid ADA results tuberculous treatment may potentially be instituted early. Similarly, a higher ADA level in younger patients would improve the accuracy of TPE diagnosis. Future large prospective studies would be needed to validate the above findings.

## Abbreviations

ADA: Adenosine deaminase; AUC: Area under curve; LDH: Lactate dehydrogenase; NPV: Negative predictive value; PPV: Positive predictive value; ROC: Receiver operating characteristics; TB: Tuberculosis; TPE: Tuberculous pleural effusion.

## Competing interests

The authors declared that they have no competing interests.

## Authors’ contributions

TR contributed to study conception and design, data analysis and interpretation, as well as article drafting and revision, and served as principal author. AT contributed to study design, data interpretation, article revision and review of the final manuscript. Both authors read and approved the final manuscript.

## Pre-publication history

The pre-publication history for this paper can be accessed here:

http://www.biomedcentral.com/1471-2334/13/546/prepub
